# Renal sympathetic denervation 2024 in Austria: recommendations from the Austrian Society of Hypertension: Endorsed by the Austrian Society of Nephrology and the Working Group of Interventional Cardiology of the Austrian Society of Cardiology

**DOI:** 10.1007/s00508-024-02440-3

**Published:** 2024-09-23

**Authors:** David Zweiker, Christian Koppelstätter, Katharina Hohenstein, Irene Lang, Sabine Perl, Heiko Bugger, Mathias-Christoph Brandt, Sabine Horn, Ronald K. Binder, Bruno Watschinger, Matthias Frick, Alexander Niessner, Thomas Weber

**Affiliations:** 1 Third Medical Department for Cardiology and Intensive Care, Vienna Healthcare Group, Clinic Ottakring, Montleartstraße 36, Pavillon 29, 1160 Vienna, Austria; 2 Division of Cardiology, Medical University of Graz, Graz, Austria; 3 Division of Nephrology, Medical University of Innsbruck, Innsbruck, Austria; 4 Division of Internal Medicine, Nephrology, Medical University of Vienna, Vienna, Austria; 5 Department of Internal Medicine II, Division of Cardiology, Medical University of Vienna, Vienna, Austria; 6 Clinic II of Internal Medicine, Paracelsus Medical University Salzburg, Salzburg, Austria; 7 Department of Internal Medicine, Villach State Hospital, Villach, Austria; 8 Department of Internal Medicine II, Cardiology and Intensive Care Medicine, Klinikum Wels-Grieskirchen, Wels, Austria; 9 Department of Internal Medicine I, Academic Teaching Hospital Feldkirch, Feldkirch, Austria; 10 Second Department of Cardiology and Intensive Care Medicine, Vienna Healthcare Group, Clinic Landstraße, Vienna, Austria

**Keywords:** Resistant hypertension, Uncontrolled hypertension, Arterial hypertension, Interventional therapy, Consensus report

## Abstract

Renal sympathetic denervation (RDN) is an interventional supplement to medical treatment in patients with arterial hypertension. While the first sham-controlled trial, SYMPLICITY HTN‑3 was neutral, with improved procedural details, patient selection and follow-up, recent randomized sham-controlled trials of second-generation devices show a consistent blood pressure lowering effect of RDN, as compared to sham controls. These new data and the recent U.S. Food and Drug Administration (FDA) premarket approval of two RDN devices are the basis for the present recommendations update.

This joint position paper from the Austrian Society of Hypertension, together with the Austrian Society of Nephrology and the Working Group of Interventional Cardiology from the Austrian Society of Cardiology includes an overview about the available evidence on RDN and gives specific recommendations for the work-up, patient selection, pretreatment, procedural management and follow-up in patients undergoing RDN in Austria. Specifically, RDN may be used in clinical routine care, together with lifestyle measures and antihypertensive drugs, in patients with resistant hypertension (i.e. uncontrolled blood pressure on 3 antihypertensive drugs) and in those with uncontrolled hypertension, after adequate work-up, if institutional, patient-related and procedural conditions are fulfilled.

## Introduction

The following recommendations should guide Austrian physicians in the use of renal sympathetic denervation (RDN) in patients with different scenarios of arterial hypertension. This is an update of the previous guidelines from the Austrian Society of Hypertension from 2014 [1], as new clinical evidence about the efficacy and safety evolved [2–18], a U. S. Federal Food and Drug Administration (FDA) premarket approval for two devices was released [19, 20] and new guidance (clinical practice guidelines, position papers, consensus statements) from the European Society of Cardiology and the European Society of Hypertension were recently published [21–24].

## History of RDN

The concept of RDN stems from the fact that increased sympathetic drive is a well-known key driver in systemic arterial hypertension [25]. As early as the 1930s, a surgical procedure known as thoracolumbar splanchnicectomy was developed for patients with severe forms of arterial hypertension and showed the effect of blood pressure lowering by sympathectomy [26, 27].

The goal of RDN is the denervation of sympathetic fibers in the adventitia of the renal arterial vasculature. In the early 2000s minimally invasive catheter-based endovascular systems were developed that facilitate the ablation of renal sympathetic nerves with high efficacy and a low complication rate. The first hype of RDN started after the publication of the initial feasibility study in 2009 [28] and the first controlled study SYMPLICITY-HTN 2 in 2010 [2]. The latter had a randomized design and found a staggering reduction of office blood pressure (BP) by 32/12 mm Hg after 6 months, while there was no difference in the control group (+1/0 mm Hg). After clinical certification the procedure was included into daily clinical care in Austria and other countries. The Austrian RDN registry with up to 300 patient cases found similar reductions in office BP compared to SYMPLICITY HTN 2 [29] and recommendations from the Austrian Society of Hypertension were published in 2012 and 2014 [1, 30]. The enthusiasm for RDN was dampened after publication of the first large sham-controlled study SYMPLICITY HTN‑3 in 2014, which found merely no effect of RDN, i.e., similar reductions of BP in the RDN and in the sham groups (Table 1). These results led to a stop in the use and reimbursement for the procedure in many countries. The European Society of Cardiology (ESC)/European Society of Hypertension (ESH) guidelines for the management of hypertension published in 2018 did not recommend RDN outside of clinical trials at all (class III indication) [31].

**Table 1:** Available randomized studies investigating RDN in hypertensive patients (adapted from [22])

Trial	Year*	Ablation technology	Inclusion criteria	Sham control	Primary endpoint	N (intervention vs. control)	Results
					Time point	Number of patients screened	
***First generation studies***							
SYMPLICITY HTN‑2 [2]	2010	RF	Resistant hypertension	*No*	oSBP at 6 months	52 vs. 54	Positive
			oSBP ≥ 160 mm Hg or oSBP ≥ 150 mm Hg and diabetics			(190)	−32 vs. +1 mm Hg, *P* < 0.0001
SYMPLICITY HTN‑3 [3]	2014	RF	Resistant hypertension	**Yes**	oSBP	364 vs. 171	Neutral
			oSBP ≥ 160 and 24hSBP ≥ 135 mm Hg		6 months	(1441)	−14.1 vs. −11.7 mm Hg, *P* = 0.26
RDN-Leipzig [5]	2015	RF	Resistant hypertension	**Yes**	24hSBP	35 vs. 36	Neutral
			24hSBP 135–149 or dayDBP 90–94 mm Hg		6 months	(1006)	−7. vs. −3.5 mm Hg, *P* = 0.15
DENERHTN [4]	2015	RF	Resistant hypertension	*No*	daySBP	53 vs. 53	Positive
			24hSBP ≥ 135 or 24hDBP ≥ 85 mm Hg		6 months	(1416)	−15.8 vs. −9.9 mm Hg, *P* = 0.0329
ReSET [15]	2016	RF	Resistant hypertension, 30–70 years	**Yes**	daySBP	36 vs. 33	Neutral
			daySBP ≥ 145 mm Hg, ≥ 85% adherence		3 months	(87)	−6.1 vs. −4.3 mm Hg, *P* = 0.66
WAVE IV [9]	2018	US (externally delivered)	Resistant hypertension	**Yes**	oSBP	39 vs. 34	Neutral
			oSBP ≥ 160, 24hSBP ≥ 135 mm Hg, ≥ 80% adherence		6 months	(239)	−13.2 vs. −18.9 mm Hg, *P* = 0.181
REDUCE-HTN: REINFORCE [11]	2020	RF***	18–75 years without antihypertensives	**Yes**	24hSBP	34 vs. 17	Neutral
			oSBP 150–180, 24hSBP 135–170 mm Hg		8 weeks	(167)	−5.3 vs. −8.5 mm Hg, *P* = 0.30
***Second generation studies***							
SPYRAL HTN-OFF MED Pilot [6]	2017	RF	Mild hypertension, 20–80 years	**Yes**	24hSBP	38 vs. 42	Positive
			oSBP 150–179, oDBP ≥ 90 mm Hg and 24hSBP 140–169 mm Hg without therapy		3 months	(353)	−5.5 vs. −0.5 mm Hg, *P* = 0.041
SPYRAL HTN-ON MED Proof of Concept [8]	2018	RF	1–3 antihypertensives, 20–80 years	**Yes**	24hSBP	38 vs. 42	Positive
			oSBP 150–180, oDBP ≥ 90 mm Hg and 24hSBP 140–169 mm Hg		6 months	(467)	−9 vs. −1.6 mm Hg, *P* = 0.005
RADIANCE-HTN SOLO [7]	2018	US	0–2 antihypertensives, 18–75 years	**Yes**	daySBP	74 vs. 72	Positive
			oSBP < 179, oDBP < 109 mm Hg and daySBP 135–179, dayDBP 85–104 mm Hg		2 months	(803)	−8.5 vs. −2.2 mm Hg, *P* = 0.0001
SPYRAL HTN-OFF MED Pivotal [10]	2020	RF	20–80 years without antihypertensives	**Yes**	24hSBP	166 vs. 165	Positive
			oSBP 150–179, oDBP ≥ 90 mm Hg und 24hSBP 140–169 mm Hg		3 months	(1519)	−4.7 vs. −0.6 mm Hg, *P* = 0.0005
RADIANCE-HTN TRIO [12]	2020	US	Resistant hypertension, 18–75 years	**Yes**	daySBP	69 vs. 67	Positive
			oSBP ≥ 140 and oDBP ≥ 90 mm Hg		2 months	(989)	−8.0 vs. −3.0 mm Hg, *P* = 0.022
			24hSBP ≥ 135 and 24hDBP ≥ 85 mm Hg under standard antihypertensive therapy				
REQUIRE [16]	2021	US	Resistant hypertension, 20–75 years	**Yes**	24hSBP	69 vs. 67	Neutral
			SBP ≥ 150 or oDBP ≥ 90 mm Hg, and *24hSBP ≥ 140 mm Hg		3 months	(411)	−6.6 vs. −6.5 mm Hg, *P* = 0.971
SPYRAL HTN ON-MED [13] **	2022	RF	1–3 antihypertensives, 20–80 years	**Yes**	24hSBP	30 vs. 32	Positive
			oSBP 150–180, oDBP ≥ 90 mm Hg and 24hSBP 140–169 mm Hg		36 months	(467)	−18.7 vs. −8.6 mm Hg, *P* = 0.0039
SPYRAL HTN ON-MED pivotal long term [17]	2023	RF	1–3 antihypertensives, 20–80 years	**Yes**	Nighttime SBP	20 vs. 18	Positive
			oSBP 150–180, oDBP ≥ 90 mm Hg and 24hSBP 140–169 mm Hg		36 months		−20.8 vs. −7.2 mm Hg, *P* = 0.001
RADIANCE II [14]	2023	US	0–2 antihypertensives, 18–75 years	**Yes**	Daytime SBP	150 vs. 74	Positive
			oSBP 140–179, oDBP 90–119 mm Hg, daySBP 135–179, dayDBP 85–104 mm Hg without antihypertensive therapy		2 months	(1038)	−7.9 vs. −1.8 mm Hg, *P* < 0.001
SPYRAL HTN ON-MED pilot + expansion study [18]	2023	US	1–3 antihypertensives, 20–80 years	**Yes**	24hSBP	192 vs. 116	Neutral
			oSBP 150–180, oDBP ≥ 90 mm Hg and 24hSBP 140–169 mm Hg		6 months	(1780)	−6.5 vs. −4.5 mm Hg, *P* = 0.12 (differences in medication use favored RDN with a win ratio 1.5 *p* = 0.005)

*oSBP* systolic office blood pressure, *oDBP* diastolic office blood pressure, *24hSBP* systolic 24-hour blood pressure, *24hDBP* diastolic 24-hour blood pressure, *daySBP* systolic daytime blood pressure (from ABPM), *dayDBP* diastolic daytime blood pressure (from ABPM), *RF* radiofrequency, *US* ultrasound, *ABPM* ambulatory blood pressure monitoring, *RDN* Renal sympathetic denervation

* year of publication, ** extension trial of the SPYRAL HTN ON-MED proof of concept study. Please refer to the text concerning the definition of resistant hypertension, *** Vessix multielectrode device

Later studies found regression to the mean, asymmetric data handling and a motivation towards better adherence to antihypertensive drugs in the RDN group to be the main drivers for positive results of the first studies [32]. These considerations, based on results from the SYMPLICITY-HTN 3 study and other first-generation sham-controlled clinical trials, led to a complete rethinking of procedural and patient-related aspects of RDN. The following advances were made in second-generation studies:

- Better screening of study patients by using ambulatory BP monitoring and regular adherence checks before and after the intervention in both the RDN and the sham group.
- Increased efficacy of RDN by using multielectrode second-generation devices, optimization of perioperative workflows and adequate training of operators.
- Exclusion of unintended bias by use of low-noise outcome variables (such as ambulatory BP instead of office BP) and performance of a blinded sham procedure in the control group.

Second-generation trials with sham-controls now paint a homogeneous picture regarding the efficacy of RDN in patients with arterial hypertension (Table 1). In patients with mild, moderate and resistant hypertension, a consistent reduction of ambulatory BP is shown compared to sham 2–36 months after the procedure. On the basis of these data RDN is clearly a suitable option for BP lowering in selected patients with arterial hypertension, in accordance with the recently published guidelines for the management of arterial hypertension by the ESH [21].

## Methods of RDN

Currently, two different physical principals are mainly used for RDN: radiofrequency (RF) ablation and ultrasound (US) ablation.

The first available system on the market was based on RF [28]. The Symplicity Renal Denervation System® (Medtronic, Minneapolis, MI, USA) has first been developed to perform point-by-point ablation around the renal artery. The first generation was time-consuming and it was deemed less efficacious because only the proximal parts could be ablated. The second generation (Symplicity Spyral, Medtronic) enables the simultaneous ablation of several points of the renal arterial system in a spiral configuration as well as an ablation of the branches of the main renal artery.

The Paradise Ultrasound Denervation System (Recor Medical Inc, Palo Alto, CA, USA) is available for RDN using unfocused US. This enables a homogeneous penetration and 360° ablation of the perivascular tissue.

Based on the favorable results from the second-generation trials outlined above, both the RF [19] and the US [20] devices received FDA premarket approval for clinical use in November 2023.

In Austria, RDN will be reimbursed in 2025 on a preliminary basis (NUB, new investigation and treatment methods, *NUB—neue Untersuchungs- und Behandlungsmethoden*).

An externally delivered US device has been explored but failed to show valid data on blood pressure lowering [9].

Other methods for RDN are under investigation. The RDN using perivascular alcohol injection has been explored in early studies, which showed promising results [21, 33]. A sham-controlled second-generation study has recently been published; the results were neutral [34]. Currently, no final recommendation can be given regarding this method.

## Safety

The RDN is an invasive, preventive procedure that causes a clear reduction in BP as a surrogate marker but no direct reduction of cardiovascular outcomes has yet been shown. Consequently, RDN has to be a proven low-risk procedure to be accepted as an alternative to antihypertensive medication, which serves as the gold standard for treatment of arterial hypertension and is highly effective with a low risk profile.

Safety data from multiple randomized controlled trials [2–16] and long-term registries [29, 35] are available. If performed by experienced operators RDN can generally be considered a safe procedure. The most common complications are access site-related and occur in around 1% of cases. They can be avoided by a US-guided puncture and/or the use of a vascular closure device. The radiation dose varies and data on long-term problems due to radiation are missing as well as data on long-term negative effects due to anesthesia. The risk of contrast-associated acute kidney injury can be prevented by balanced hydration. Major vascular complications other than at the access site (i.e., dissection, perforation, intrarenal hematoma) are conceivable in theory but exceedingly rare events. In a meta-analysis of 50 trials and 5769 patients undergoing RF RDN, 7 intraprocedural dissections resulting in stent implantations were reported [36]. In general, these acute complications can be avoided with proper RDN techniques [22]. Previously feared long-term complications of RDN, such as decline in renal function and renal artery stenosis have not been shown in large observational studies and controlled trials [10, 36]. In a meta-analysis, renal arterial long-term complications (i.e., new renal artery stenoses) occurred with a similar incidence as in an untreated hypertensive population [22]. In another meta-analysis it was concluded that renal function did not change significantly for at least 9 months after RDN [37].

## Efficacy of RDN

Current data show that, compared to sham-group patients, RDN leads to a moderate, albeit clinically significant reduction in 24 h SBP of 4–7 mm Hg in nearly all patient groups, from hypertension grade I to resistant hypertension. A reduction in inpatient admissions due to hypertensive emergencies was also recorded after RDN [38]; however, the procedural success is dependent on specific patient-related and procedure-related factors, which are discussed in the following sections. The durability of RDN is still a theoretical question as preclinical data suggest that reinnervation may occur theoretically 30 months after RDN in a mouse model [39]. Long-term randomized clinical trials, however, indicate a consistent BP reduction with a trend to lower BP over time for at least 3 years [13]. Recently, long-term follow-up studies of 9 and 10 years after RDN became available, indicating a long-lasting BP lowering effect of the procedure [40, 41].

Based on registry data, the reduction in BP is independent of the number and class of antihypertensive drugs at baseline [42]. Furthermore, after 3 months follow-up more patients decreased than increased the number of antihypertensive drugs.

## Center qualification

First-generation randomized controlled trials of RDN showed that the procedure can lead to suboptimal results and a higher risk of complications in inexperienced centers with a low case load. For example, in the SYMPLICITY-HTN 3 study [3], low operator experience and ineffective RDN have been discussed as probable reasons for the neutral outcome [43]. Nonstandardized patient pathways can lead to inadequate patient selection without guideline-directed medical treatment or exclusion of secondary hypertension. Patients with unsuitable anatomy have to be identified preprocedurally. Furthermore, institutional expertise to adequately treat rare complications has to be present, especially in vascular surgery. The importance of institutional experience regarding RDN is highlighted in the ESH guidelines, which also recommend limiting the use of RDN to experienced centers [21].

*The* *RDN centers should have a dedicated hypertension outpatient department, inpatient ward and departments of radiology, cardiology, nephrology, laboratory diagnostics, on-site vascular surgery and a coronary/intensive care unit. Specialization for management of complex patients with arterial hypertension is necessary and can be evidenced with dedicated diplomas (Hochdruckspezialist Österreichische Gesellschaft für Hypertensiologie, European Specialist in Hypertension ESH, Excellence Center ESH).*

## Multidisciplinary hypertension team (MDT)

A multidisciplinary hypertension team (MDT) can be formed that enables the informed discussion of patients suitable for RDN from various viewpoints. We strongly recommend that a hypertension specialist, certified by the ESH or a “Hochdruckspezialist” certified by the Austrian Society of Hypertension takes part in hypertension team meetings. In addition to RDN operators, a clinical cardiologist, a nephrologist and a specialist experienced in sedation (e.g., anesthesiologist, intensive care specialist) should participate. This multidisciplinary approach is also endorsed by the ESH guidelines (class I recommendation [21]).

*The final decision to perform RDN should be made by a dedicated multidisciplinary hypertension team that includes at least a certified hypertension specialist, an RDN operator, a clinical cardiologist, a nephrologist and an expert on analgosedation.*

## RDN operators

The RDN operators should be experts in percutaneous cardiovascular interventions including access site management, radioprotection, periprocedural BP management, analgesia, and the renal arterial anatomy. We recommend that operators should first gain experience in vascular interventions before performing RDN. Furthermore, operators should receive hands-on training using a bench model of RDN and off-site attendance in an active RDN center. Proctoring of the first cases should reduce the risk of complications in operators starting to become self-dependent.

*The RDN operators should have performed a sufficient number of RDN procedures with a proctor before performing an RDN procedure independently. To retain experience, operators should perform RDN procedures on a regular basis.*

## Patient selection

### Adherence to medical treatment

Ensuring adherence to medical treatment is one of the cornerstones of initially asymptomatic diseases, such as hypertension [44]. Current guidelines for treatment of hypertension recommend the use antihypertensive polypills to increase adherence by reduction of side effects and ease of use [44]. An informed discussion with the patient about possible side effects is essential when starting antihypertensive treatment. The time of drug intake, in the morning or in the evening, may be adapted to best fit the daily life of the patient as the TIME study did not show any benefit of the evening over the morning dose administration [45]. Good adherence leads to better outcomes [46] but assessment of adherence is challenging outside clinical trials and may be very difficult to measure in the clinical routine [21]. The adherence to antihypertensive medication should be checked and discussed with the patient. In a large number of patients as evidenced for instance in all recent high-quality RDN trials, persistent and complete adherence is hard to achieve. As the primary goal is BP control, patients who are repeatedly nonadherent (if this reflects the unwillingness of the patient to take drugs) or intolerant to multiple antihypertensive drugs, can also be considered for RDN after information about the potential lack of effect and benefits and also the risks associated with the procedure. These patients may be on fewer than three drugs at the time of their selection for RDN [21, 22].

*Adherence to medical treatment should be ascertained before considering RDN in patients with arterial hypertension, for instance with witnessed drug intake, laboratory drug monitoring, or monitoring of prescription refills. The results of these tests should be discussed with the patient. As BP lowering is the goal to reduce cardiovascular risk, RDN could be an option for patients unable to be fully adherent to antihypertensive drugs, for instance due to side effects, in certain conditions.*

### Screening for RDN and shared decision making

All patients considered for RDN have to undergo investigations, screening for secondary hypertension as recommended by international guidelines [21] and optimization of treatment at a hypertension clinic. The 24 h ambulatory BP monitoring (ABPM) is an integral part of the diagnostic work-up to exclude white-coat hypertension.

*Before RDN can be considered, secondary hypertension has to be excluded, antihypertensive treatment should be optimized at a hypertension clinic, and persistence of high BP has to be evaluated using ABPM* *.*

As RDN is an invasive procedure, available and safe oral reserve antihypertensive medications as possible alternatives, potential complications and the need to continue medical antihypertensive treatment despite the procedure in most instances, should be discussed thoroughly with the patient. In addition to individual clinical expertise and available external clinical evidence from high-quality RDN studies [47], the patient’s specific needs as well as possible intolerances to medical treatment, should be incorporated in the final decision to perform RDN.

*The patient’s needs and expectations should be included in the final decision to perform RDN.*

### Resistant hypertension

Resistant hypertension is defined as not reaching BP targets despite treatment with at least three antihypertensive medications including one diuretic at maximum tolerated doses [44]. Patients with resistant hypertension are the best studied hypertensive population undergoing RDN and are therefore the preferred patient group. We propose the following inclusion criteria for patients undergoing RDN:

*The RDN is a reasonable additional treatment option in patients with resistant hypertension and:*

- *Taking at least 3 different antihypertensive medications, one of which should be a diuretic.*
- *Have an average 24‑h SBP of ≥* *130* *mm* *Hg or an average daytime SBP of ≥* *135* *mm* *Hg in a recent 24‑h BP recording.*
- *Are at least 18 years old.*
- *Have an estimated glomerular filtration rate of ≥ 40 ml/min/1.73 m* ^*2*^ *body surface area.*

### Mild hypertension

In patients with arterial hypertension grade I who take only few antihypertensives (usually defined as 0–1 antihypertensives), RDN may prevent the necessity of taking antihypertensives at all. Two studies found positive results of RDN in this patient population [6, 10]; however, a high number of different well-tolerated and well-studied antihypertensive medications are available for this patient population. Therefore, the panellists believe that RDN may only be considered in this patient population in selected cases after carefully evaluating benefits and harms, especially taking the individual tolerability to antihypertensive medication into account.

*In patients with mild hypertension, RDN may be considered in selected cases, specifically in the presence of intolerance to several antihypertensive drug classes, considering the patient’s needs and shared decision making.*

### Uncontrolled hypertension with intolerance to antihypertensive drugs

Patients with uncontrolled hypertension have been included in several sham-controlled trials with improvement in BP control [8, 14]. The use of RDN may therefore be considered an option for patients with uncontrolled hypertension despite attempting lifestyle modifications and antihypertensive medication but who are either intolerant to additional medication or do not wish to be on additional medications and who are willing to undergo RDN after shared decision-making [48].

*The RDN can be considered as a treatment option in patients with an eGFR >* *40* *ml/min/1.73 m*^*2*^* who have uncontrolled BP, if drug treatment elicits serious side effects and poor quality of life.*

## Current European Society of Hypertension guidelines

In 2023 the European Society of Hypertension released new guidelines incorporating RDN as a treatment option in patients with an eGFR of > 40 ml/min/1.73 m^2^ with uncontrolled BP despite the use of antihypertensive drug combination treatment or if drug treatment would lead to serious side effects and reduced quality of life (class of recommendation II, level of evidence B) [21]. Furthermore, RDN can be considered as an additional treatment option in patients with true resistant hypertension and eGFR > 40 ml/min/1.73m^2^ (class of recommendation II, level of evidence B); however, RDN should only be performed in experienced specialized centers and the selection of patients undergoing RDN must incorporate shared decision making (class of recommendation I for both recommendations, expert opinion) [21].

## Predictor of response to RDN

Although RDN undoubtedly lowers BP in groups of patients, the effect of the intervention in individual patients is heterogeneous, resembling the situation with different antihypertensive drug classes [49]. The topic is currently under investigation and potential candidates are heart rate [50], pulsatile hemodynamics/arterial stiffness [51], renin [52], and many others.

## When not to perform RDN

**Fig. 1:**
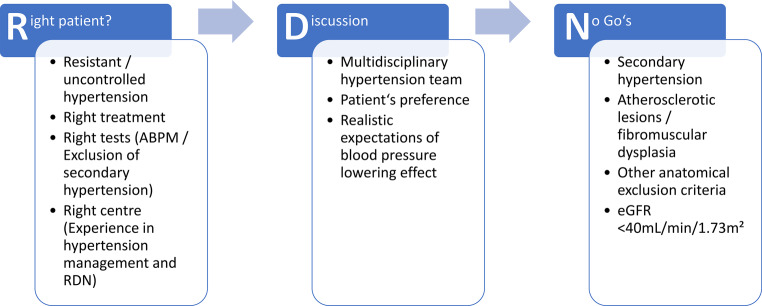
Main principles in selecting the right patients for RDN. *RDN* renal denervation

Due to technical or individual considerations or absence of evidence, patients with the following factors should not undergo RDN (Fig. 1).

*The RDN should not be performed in patients with the following prohibitive conditions (contraindications):*

- *Unsuitable renal arterial anatomy.*
  - *Presence of accessory untreatable arteries.*
  - *Inappropriate vessel diameter.*
  - *Advanced renal artery atherosclerosis.*
  - *Renal artery stenosis.*
  - *Fibromuscular dysplasia.*
  - *Previous renal artery stenting.*
- *Secondary hypertension.*
- *Undergoing abdominal dialysis or hemodialysis.*
- *Unstable clinical situations (acute coronary syndromes, acute cerebrovascular events etc.).*
- *Pregnancy.*
- *Age <* *18 years or >* *85 years.*

*The RDN should not be performed in patients in the following situations due to insufficient clinical evidence:*

- *Severely impaired kidney function (<* *40* *mL/min).*
- *Single functioning kidney.*
- *Kidney transplant recipients.*

## Procedural considerations

### Procedural planning and patient preparation

Adequate imaging is crucial for procedural planning and identification of potential anatomical ineligibilities.

*Non-invasive renal artery imaging using either computed tomography or magnetic resonance imaging should be preferred over duplex ultrasound to identify:*

- *The presence of accessory arteries.*
- *Anatomical anomalies that prohibit an RDN procedure (e.g., inappropriate vessel diameter, untreated atherosclerotic or fibromuscular dysplasia, renal artery stenosis).*
- *Extent of abdominal aorta/iliofemoral arteries atherothrombotic disease.*

*Selective renal angiography immediately before RDN remains the gold standard for identification of renal artery abnormalities.*

To reduce complications all measures should be undertaken to minimize the risk of complications. This includes adequate preparation of the procedure as well as sophisticated bail-out strategies. The following recommendations are adapted from the clinical consensus statement from the ESC Council on Hypertension and the European Association of Percutaneous Cardiovascular Interventions regarding renal denervation in the management of hypertension in adults [22].

- *We recommend the establishment of a standard operating procedure that includes acute management in case of complications.*
- *Continuous monitoring of vital parameters should be performed to identify complications early.*
- *If applicable, antidotes of anaesthetics should be available in the catheter laboratory (e.g., naloxone and flumazenil).*
- *Patients should be hydrated to euvolemia to reduce the risk of acute kidney injury.*
- *Intraprocedural administration of unfractionated heparin (100* *U/kg or a target ACT >* *250* *s) is advised.*
- *Preprocedural aspirin should be administered as loading dose, followed by 100* *mg daily until 1 month postprocedure. In the case of oral anticoagulant therapy, antithrombotic therapy should be tailored according to ESC guidelines for chronic coronary syndromes related to endovascular interventions* [53].

### Procedure

As ablation of the renal arteries is painful, patients should be sedated during the procedure by a specialist trained in sedation. Analgesia may be performed with opioids. Vital signs should be monitored and intravenous drugs for BP control should be available in the catheter laboratory.

*For RDN we recommend analgosedation with low doses of opioids (e.g., fentanyl) together with sedating drugs (e.g., midazolam or propofol).*

*Intra-arterial nitrates are recommended preprocedurally (in the absence of hypotension). The BP should be monitored invasively and corrected when necessary. Intravenous drugs for BP control should be available in the catheter laboratory.*

As a significant proportion of complications are derived from the vascular access, it should be gained with maximum caution and all available tools should be used to minimize the risk of adverse events. Radiation should be kept to a minimum. A 6 French catheter is used in RF ablation and a 7 French catheter in the US-based device.

*Femoral arterial access may be performed under US guidance, if the operator is experienced to do so. Vascular closure devices should be used to reduce the risk of complications.*

*Modern monoplane or biplane angiographic systems should be used to reduce the radiation dose.*

*At the end of the procedure, angiography of the renal artery should exclude potential renal parenchymal or arterial injuries.*

## Follow-up and quality control

Regular follow-up of patients undergoing RDN is necessary to monitor and eventually react to changes in BP profiles or renal function. By regular follow-up long-term complications can be identified and treated earlier. At the suspicion of a late renal vascular complication, renal angiography by computed tomography or vascular ultrasound should be performed.

*Centers performing RDN are responsible for adequate follow-up at 3, 6 and 12 months after the procedure and at yearly intervals thereafter, including assessment of renal function and BP.*

While RDN shows consistent BP reduction in selected patients in the setting of randomized controlled trials in experienced centers, there are still limited data about the use of second-generation devices in daily clinical practice in centers with less experience. It is therefore crucial to document the efficacy and safety of RDN outside of clinical trials. This documentation furthermore serves as quality control for centers performing RDN. A national prospective registry [29, 54] will be re-established that should capture baseline, procedural and outcome data of all RDN procedures in Austria.

*Procedural and outcome data of all patients undergoing RDN should be collected and included* *in a prospective trial, study and/or registry.*

## Conclusion

This position paper should be seen as guidance for physicians performing RDN in Austria. When specific conditions regarding the RDN center, the patient and the procedure are fulfilled, RDN can be a useful supplement to medical antihypertensive treatment in patients with arterial hypertension.
